# Perceptions of Emergency Medical Care students on high-fidelity manikins versus standardised patients: Insights from a pilot mixed-methods study

**DOI:** 10.4102/hsag.v31i0.3306

**Published:** 2026-05-30

**Authors:** Karien Henrico, Getrude M. Motadi

**Affiliations:** 1 Department of Emergency Medical Care, Faculty of Health Sciences, University of Johannesburg, Johannesburg, South Africa

**Keywords:** student satisfaction, standardised patient, high-fidelity manikin, simulation-based education, South Africa

## Abstract

**Background:**

Simulation-based education is central to Emergency Medical Care (EMC) training, particularly in preparing students for high-pressure, pre-hospital environments. High-fidelity manikins (HFMs) and standardised patients (SPs) are widely used simulation modalities; however, comparative evidence from low- to middle-income countries remains limited. Understanding student satisfaction with these modalities is important for ensuring educational quality and making informed resource allocation.

**Aim:**

This study explored EMC students’ satisfaction with HFMs and SPs to inform pedagogical and resource-related decisions.

**Setting:**

The study was conducted in a university-based simulation laboratory at a South African higher education institution.

**Methods:**

A pilot mixed-methods design was employed. Fifteen first-year EMC students completed the same clinical simulation scenario using both a SP and a HFM. Satisfaction was measured using a revised Satisfaction Scale for High-Fidelity Clinical Simulation and two open-ended questions. Quantitative data were analysed descriptively, and qualitative responses were analysed thematically.

**Results:**

High levels of satisfaction were reported for both simulation modalities. Standardised patients received higher satisfaction scores for realism, theory-practice integration and self-confidence, while HFMs were rated more favourably for practising technical skills and learning from mistakes. Qualitative findings indicated that SPs enhanced realism and engagement but were associated with increased stress.

**Conclusion:**

Despite the small cohort, clear preferences and educational implications emerged. The findings support the use of blended simulation approaches and highlight the need for further, larger-scale research.

**Contribution:**

This study provides preliminary evidence to inform simulation-based curriculum design and resource allocation in EMC education within resource-constrained contexts.

## Introduction

Simulation-Based Education (SBE) has become a critical pedagogical tool in health professions education, providing a structured and safe learning environment that facilitates the acquisition of clinical skills, critical thinking and confidence among healthcare students (Arrogante et al. 2021). Defined as a teaching method that replicates clinical scenarios to allow learners to practise and perfect their skills without causing risk to patients, SBE is especially valuable in settings where clinical exposure is limited or irregular (Aebersold 2018; Rutherford-Hemming et al. 2019). Educators see SBE as a cornerstone of health professions training, as it offers a structured environment to integrate theoretical knowledge with clinical practice. By recreating real-world scenarios, SBE allows students to practise patient management, develop decision-making skills and build confidence without risk to actual patients (Alconero-Camarero et al. 2016; Shankar et al. 2016). Prehospital emergency care, in particular, benefits from SBE as it prepares students for unpredictable and high-pressure scenarios (Slabber & Henrico 2022).

Fidelity in SBE refers to the extent to which a simulated experience reflects real clinical practice (Lateef 2010). Low- and medium-fidelity simulations focus on limited or partially interactive features, whereas high-fidelity simulations employ advanced manikins with dynamic physiological responses (Lateef 2010). The fidelity used during SBE should rely on the educational objectives and student level. High-fidelity simulations often involve High-Fidelity Manikins (HFMs) and Standardised Patients (SPs), each offering distinct advantages.

High-Fidelity Manikins offer technological precision in reproducing physiological signs and procedural feedback, ideal for practising technical skills. Standardised patients involve trained individuals who simulate patient encounters to support realistic interaction and clinical reasoning (Barrows 1993). Standardised patients provide the nuanced realism of human interaction, enabling students to build communication, empathy and patient-centred care competencies (Cupic 2018; Myung et al. 2010). Despite their complementary roles, few studies in the South African context have examined how learners perceive these modalities comparatively, particularly within undergraduate Emergency Medical Care (EMC) programmes. Emergency Medical Care students are expected to perform under high-pressure conditions and make rapid, autonomous clinical decisions, skills that SBE is uniquely suited to develop (Van Wyk, Labuschagne & Joubert 2020).

Although studies have evaluated each modality independently, there is a lack of comparative research contextualised in low- to middle-income countries (LMICs) like South Africa, where resource allocation is a major consideration in educational planning. Understanding students’ preferences and satisfaction is crucial not only for improving learning outcomes but also for guiding efficient use of institutional resources. This pilot study aims to explore how undergraduate EMC students perceive and compare HFMs and SPs in a controlled simulation setting. It provides insight into the value of each modality in shaping foundational clinical competence and informs local curriculum development, where resource optimisation is critical.

This study was designed as a pilot investigation to explore EMC students’ satisfaction with SPs and HFMs in SBE. The aim was to generate preliminary insights and assess the feasibility of a future, larger-scale study.

### Theoretical framework

This study is underpinned by Kolb’s Experiential Learning Theory, which provides a theoretical lens for understanding how students engage with simulation-based learning. Kolb’s theory conceptualises learning as a dynamic process that integrates experience, reflection, understanding and action (Shanthi & Malathi 2024). Simulation aligns with this theory by offering learners a controlled environment to experience clinical scenarios, reflect on performance, integrate theoretical understanding, and apply this in future situations.

Kolb’s Experiential Learning Theory was used to frame how students engage with simulation as an experiential process. Both SPs and HFMs support experiential learning, although they may emphasise different aspects of engagement, interaction and skill application. By comparing these two modalities, the study explores how each contributes to different stages of learning for early-year EMC students.

## Research methods and design

### Study design

This study employed a quantitative comparative design, supplemented with qualitative data from open-ended responses. This mixed-methods approach was selected to provide both measurable outcomes and deeper insights into student perceptions. The design enabled comparison of satisfaction levels across two simulation modalities (SPs and HFMs) within the same clinical scenario.

### Setting

Data collection took place in a university-based simulation laboratory in South Africa. This space is equipped with both high-fidelity simulation tools (e.g. Sim-Man 3G) and environments suitable for SP interactions.

### Study population and sampling justification

No formal sample size calculation was undertaken, as the study was intentionally designed as a pilot. The sample consisted of all eligible and consenting first-year EMC students to explore feasibility and preliminary trends rather than achieve statistical power. First-year students were included to explore early perceptions of simulation modalities before extensive clinical or simulation exposure, which aligned with the pilot design of the study. At the time of data collection, the study population consisted of 38 first-year students who were enrolled in the EMC I theory and practical module (*N* = 38). A convenience sample consisted of 15 students who freely participated in the study (*n* = 15). All participants were above the age of 18 years and had received prior orientation to the simulation environment. While the small sample size limits generalisability, it remains valuable for exploratory and pilot purposes.

### Intervention

Participants completed the same simulation scenario twice, once using an SP and once using an HFM. The scenario involved the diagnosis and stabilisation of a tibia-fibula fracture following a pedestrian-vehicle accident. This case was chosen for its feasibility, clinical relevance and reproducibility across both simulation modalities. All participants completed the simulation scenarios using SP first, followed by HFM. Randomisation of simulation modality order was not undertaken because of logistical constraints.

The scenario included a standard pre-brief, consistent instructions, and access to identical simulation equipment in both sessions. Students were evaluated individually, without team interaction. No formal debriefing was conducted in this study. This was an intentional methodological decision aimed at capturing students’ immediate perceptions of the simulation modality itself, rather than reflections influenced by facilitator-led discussion or peer feedback.

### Data collection

A revised version of the Satisfaction Scale for High-Fidelity Clinical Simulation (SSHF) was used in this study (Jiménez-Álvarez et al. 2024). This 28-item Likert-type questionnaire measures eight domains, including simulation utility, case realism, self-confidence, theory-practice integration and perceived learning. Domain 3, focused on debriefing and team communication, was removed as the study focused only on individual performance without debriefing. Two open-ended questions were added:

1. *How would you describe this clinical simulation experience?*
2. *Which of the two modalities did you find most useful, and why?*

The questionnaire was completed electronically following the simulation sessions, and responses were submitted anonymously with no identifying information collected.

### Data analysis

Quantitative data were analysed using Microsoft Excel (Microsoft Corporation 2021).

Descriptive statistics (mean and standard deviation) were used to assess scores across domains and modalities. Open-ended qualitative responses were analysed thematically to identify common patterns.

The SSHF tool used had been previously validated (Alconero-Camarero et al. 2016). The 28-item SSHF questionnaire was analysed across seven domains. The five-point Likert-scale responses were collapsed into three categories to enhance interpretability, given the small sample size. These categories comprised disagreement (strongly disagree and disagree), neutral (indifferent) and agreement (agree and strongly agree). Likert-scale data were treated as ordinal data. Inferential statistical testing (such as the Wilcoxon signed-rank test) was not undertaken given the exploratory nature of the pilot study and limited sample size; such analyses are planned for future, larger-scale studies.

Open-ended qualitative responses were analysed thematically to identify common patterns.

Coding was done manually using an inductive thematic approach. No qualitative data analysis software was used given the small volume of qualitative data. Responses were read repeatedly, coded, and grouped into themes based on recurring patterns in the data. Initial coding was followed by categorisation into major themes such as realism, stress, communication and procedural confidence. Data saturation was not sought, as the qualitative component was exploratory and intended to supplement the quantitative findings in this pilot study. Qualitative findings were used to provide contextual depth to the quantitative results rather than to develop a theory.

### Ethical considerations

Ethical approval was granted by the Faculty of Health Sciences Undergraduate Research Ethics Committee (REC-112-2022). Institutional and departmental permissions were obtained. Participation was voluntary, anonymity was ensured, and participants were informed of their right to withdraw from the study at any time. Written consent was given if participants wanted to participate in the study. Written informed consent was obtained from all individual participants involved in the study. Anonymity was ensured by collecting no identifying information, and questionnaire responses could not be linked to individual participants. Ethical approval was obtained through institutional processes, and all data collection adhered to principles of confidentiality and participant safety.

## Results

A total of 15 EMC students participated in this study. All were enrolled in the same academic module and had completed introductory simulation training as part of their curriculum. To minimise the risk of identifying participants within the small sample, demographic information was not gathered. The sample included both male and female students, with all participants aged 18 years or older.

### Quantitative findings: Satisfaction scale analysis

The overall mean satisfaction score for the SPs was 121.8 (s.d. = 11.52), and for the HFMs, it was 114.3 (s.d. = 12.5). Students reported high satisfaction with both modalities, with SPs scoring higher overall.

Table 1 summarises mean scores across domains, highlighting areas where SPs outperformed HFMs. Notable differences were observed, with the SP being particularly favoured for its realism (SP mean = 4.60, s.d. = 0.63), student self-confidence (SP mean = 4.18, s.d. = 0.97) and its ability to integrate theory and practice (SP mean = 4.43, s.d. = 0.84). In contrast, the HFMs scored slightly higher in clinical competence improvement (HFM mean = 4.87, s.d. = 0.52) and allowing learners to learn from their mistakes (HFM mean = 4.87, s.d. = 0.52).

**TABLE 1:** Mean and standard deviation values of the Satisfaction Scale for High-Fidelity Clinical Simulation for standardised patients and manikin.

SSHF Item	Mean SP	s.d.	Percentage			Mean manikin	s.d.	Percentage		
			In disagreement	Indifferent	In agreement			In disagreement	Indifferent	In agreement
**Domain 1: Simulation utility**										
7. Clinical simulation is useful to assess a patient’s clinical situation.	4.33	0.62	0	7	93	4.72	0.80	0	20	80
10. Simulation has improved my ability to provide care to my patients.	4.67	0.49	0	0	100	4.33	0.62	0	7	93
12. Simulation improves communication and teamwork.	4.73	0.46	0	0	100	4.33	0.82	0	20	80
15. Simulation allows us to plan patient care effectively.	4.27	1.03	13	0	86	4.00	1.00	13	7	80
16. I have improved my technical skills.	4.60	0.51	0	0	100	4.33	0.72	0	13	87
17. I have reinforced my critical thinking and decision-making.	4.53	0.52	0	0	100	4.07	0.88	7	13	80
18. Simulation helped me to assess the patient’s condition.	4.47	0.64	0	7	93	4.33	0.72	0	13	87
19. This experience has helped me prioritise care.	4.53	0.52	0	0	100	4.20	0.68	0	13	86
21. I have improved communication with my patients.	4.60	0.51	0	0	100	4.07	0.70	0	20	80
24. Interaction with simulation has improved my clinical competence.	4.33	0.62	0	7	93	4.40	0.83	0	20	80
26. I have learned from the mistakes I made during the simulation.	4.60	0.51	0	0	100	4.87	0.52	0	7	93
**Domain 2: Characteristics of cases and applications**										
2. Objectives were clear.	4.13	0.99	7	20	74	3.80	1.15	7	33	60
5. The degree of difficulty was appropriate to my knowledge.	4.20	0.86	7	7	87	4.00	0.85	7	13	80
6. I felt comfortable and respected during the sessions.	3.93	1.22	20	13	67	3.87	1.06	7	20	74
8. Clinical simulation helps you to learn to avoid mistakes.	4.33	0.90	7	7	86	4.27	0.96	7	13	80
**Domain 4: Self-reflection on performance**										
9. Simulation has helped me to set priorities for action.	4.47	0.64	0	7	93	4.33	0.62	0	7	93
11. Simulation has made me think about my next clinical practice.	4.40	0.91	7	7	87	3.93	0.80	7	13	80
27. The practicality of the simulation modality.	4.07	0.96	0	40	60	3.73	0.80	0	47	53
**Domain 5: Increased self-confidence**										
20. Simulation promotes self-confidence.	4.13	1.25	14	7	80	3.80	1.15	14	13	74
22. This type of practice has increased my ability to treat a real-life person.	4.33	0.82	0	20	80	4.13	0.83	0	27	73
28. Overall satisfaction with the scenario.	4.20	0.86	0	27	74	3.87	0.74	0	33	67
**Domain 6: Relations between theory and practice**										
3. The case recreated a real situation.	4.60	0.63	0	7	94	4.00	0.85	0	27	74
4. The timing of the simulation has been adequate.	4.13	1.06	7	27	66	3.87	1.06	7	40	53
14. Simulation is beneficial to relate theory to practice.	4.60	0.74	0	13	86	4.40	0.83	7	0	93
**Domain 7: Facilities and equipment**										
1. The facilities and equipment were real.	4.53	1.06	7	0	93	3.87	1.30	27	7	67
23. I lost calm during the simulation.	3.93	1.03	7	33	60	3.47	1.06	20	33	47
25. I knew the theoretical aspect of the scenario.	3.73	1.16	14	20	67	3.67	1.23	20	13	67
**Domain 8: Negative aspects of simulation**										
13. Simulation has made me more aware/worried about clinical practice.	4.40	0.63	0	7	94	4.07	0.80	0	27	73

SSHF, Satisfaction Scale for High-Fidelity Clinical Simulation; SP, standardised patients; s.d., standard deviation.

It should be noted that a higher mean score for each scale or question indicates a higher level of agreement with it (Alconero-Camarero et al. 2016; Jiménez-Álvarez et al. 2024). The SSHF is divided into eight domains; however, the third domain was excluded because of the specific focus of this study (Table 2). As per the instructions from the survey developers, the questions were grouped to form these domains. The mean value for all SP domains was above or equal to four.

**TABLE 2:** Mean and standard deviation values for simulation modality type per domain.

SSHF domain	SP		Manikin	
	Mean	s.d.	Mean	s.d.
1. Simulation utility	4.97	0.60	4.72	0.77
2. Characteristics of cases and applications	4.15	0.99	3.98	1.00
3. Communication	-	-	-	-
4. Self-reflection on performance	4.31	0.85	4.00	0.77
5. Increased self-confidence	4.18	0.97	3.73	0.91
6. Relationship between theory and practice	4.43	0.84	4.09	0.90
7. Facilities and equipment	4.07	1.12	3.67	1.10
8. Negative aspects of the simulation	4.40	0.63	4.07	0.80

SSHF, Satisfaction Scale for High-Fidelity Clinical Simulation; SP, standardised patients; s.d., standard deviation.

For the manikin, only ‘Increased self-confidence’ (3.98) and ‘Facilities and equipment’ (3.67) were below four, as seen in Table 2.

### Qualitative findings: Open-ended responses

The analysis of responses to the open-ended questions yielded three recurring themes: nervousness, realism and improvement. Students noted that SPs heightened realism but also increased stress levels.

Participants in this study seemed to appreciate SP more, but they also mentioned that it added to their stress levels or nervousness during the simulation. This is seen in the following quotes:

‘Since it’s a real patient, I think of the mistakes I could make which makes me nervous. It’s hard to critically think since the patient needs help at that moment so I usually have the mentality that everything has to be done in a rush.’ (Participant 6)

‘I would describe it as nerve-wracking because when you are doing a sim all the knowledge you have sort of goes away due to being nervous. For me, I would say I get scared at the beginning of the sim but as time goes by, I calm down and get my system in place. Things go south if I do something and the patient deteriorates, I lose my cool and I just panic but overall doing sim on a manikin helps me learn different ways of treating the patient and I learn to fix my mistakes.’ (Participant 14)

Although students expressed heightened stress during SP scenarios, they acknowledged that this stress mirrored real clinical pressures. They appreciated the interactive nature of SPs and felt more ‘invested’ in patient outcomes:

‘I was nervous treating a real person, which pushed me to think more critically.’ (Participant 1)

‘The mannequin is helpful, but the SP gave me a better understanding of real-life urgency.’ (Participant 7)

Participants appreciated the opportunity to perform simulation scenarios using the SP modality because it helped them to improve their communication and other skills, as seen in the excerpt below:

‘The clinical simulation experience has been very educational and interesting. I feel that it has helped me prepare for the real clinical environment in a calm and restricted environment where I can make mistakes and learn from my peers’ mistakes also.’ (Participant 10)

Participants felt more exposed to the real environment when doing the simulation scenario using the SP modality:

‘I believe it was very helpful in understanding how to treat patients in reality. However, some of the interaction with a manikin does not reflect reality and makes it difficult to understand.’ (Participant 12)

Most participants mentioned that doing simulations with SPs was interesting and helpful because they could easily interact with the patients, and it was more realistic than doing simulations with manikins. However, some participants were stressed during the simulation because they were scared that they might do something wrong:

‘Real person treatment is very different from practising with a manikin, so I had fears of doing something that may be detrimental to the patient or prevent the patient from recovering.’ (Participant 2)

The last question in the questionnaire asked the students to identify which of the two modalities they preferred. Twelve participants chose SPs, and only three participants preferred HFMs.

### Reliability, validity and trustworthiness

The study utilised the SSHF, a previously validated tool that is widely used in healthcare simulation research (Alconero-Camarero et al. 2016). The instrument demonstrated internal consistency in earlier studies, with Cronbach’s alpha values consistently above 0.80, indicating high reliability. In this study, reliability was supported by consistent patterns of responses across domains, with narrow standard deviations in most items. This consistency suggests that the tool yielded stable results when used with this population of EMC students.

Validity in this study was addressed through both content and construct considerations:

**Content validity:** The SSHF questionnaire covers key domains relevant to SBE, including realism, self-confidence and integration of theory with practice. The minor adaptation (removal of Domain 3, communication or debriefing) was justified given the study’s design, which excluded team-based debriefing.

**Construct validity:** The findings aligned with existing literature, which indicates that SPs are perceived as more realistic and engaging, while HFMs are valued for procedural learning (Alsaad et al. 2017; Sterz et al. 2022). This congruence with prior research supports the instrument’s ability to capture relevant constructs.

**External validity:** Although the small sample size limits generalisability, the findings may be transferable to comparable educational settings in South Africa and other LMICs where decisions regarding simulation resources are particularly important.

The trustworthiness of the qualitative component was addressed using the criteria proposed by Lincoln and Guba (1985). Credibility was supported through the inclusion of direct participant quotations to illustrate and substantiate the identified themes. These quotations were used to ensure that the findings accurately reflected participants’ experiences and perspectives.

Dependability was enhanced by clearly documenting the qualitative analysis process, including the steps followed during manual coding and theme development. Confirmability was addressed by ensuring that themes were derived directly from participants’ responses rather than from preconceived assumptions. The analysis focused on patterns evident in the data, and qualitative findings were used to complement, rather than override, the quantitative results.

Transferability was supported through a detailed description of the study context, participant group and educational setting. Although the qualitative findings are not intended to be generalisable, sufficient contextual information is provided to allow readers to assess their relevance to similar educational environments. The use of a validated measurement tool alongside thematic analysis of qualitative responses strengthens the trustworthiness of the findings.

## Discussion

This was a preliminary, single-institution study that compared student satisfaction regarding the use of SP and HFM modalities in pre-hospital emergency SBE scenarios. There was no randomisation of the simulation modalities, and the findings should therefore be interpreted as students’ perceptions of the simulation modalities rather than definitive evidence of modality-specific effects. There is no SP programme at this institution; hence, the students often practice on various fidelity manikins and are less exposed to SPs. The results demonstrated that students perceived SPs as more realistic and engaging across most domains, particularly in relation to theory-practice integration, self-confidence and overall satisfaction. High-Fidelity Manikins, however, were preferred for specific aspects, such as practising technical skills, clinical assessment and learning from mistakes.

Twelve of 15 participants indicated a preference for SPs, highlighting the perceived authenticity of human interaction. However, students also reported higher stress levels in SP scenarios, reflecting the heightened emotional and cognitive demands of engaging with real people rather than simulated technology.

This study echoes the global literature, emphasising the complementary strengths of SPs and HFMs in simulation (Alsaad et al. 2017; Sterz et al. 2022). Students favoured SPs for realism and communication, which are crucial in early EMC training; however, they also recognised the value of HFMs in practising procedures without risking harm.

Students may value the SP modality because it allows direct engagement with a patient and facilitates the active gathering of clinically relevant information (Shankar et al. 2016). Meaningful patient interaction has long been recognised as a central component of undergraduate medical education, as clinical management is grounded in information obtained through communication with the patient (Khan 2021). Such interactions enable students to develop integrated competencies in history gathering, communication, physical examination and clinical reasoning (Shankar et al. 2016).

The manikin scored higher than SP in one item that focused on improving clinical competence. In general, the use of SBE has shown a direct improvement in clinical outcomes and competence (Alconero-Camarero et al. 2016). Study participants expressed that they are afraid of making mistakes, especially when it involves someone’s life. Making mistakes often decreases students’ self-esteem and self-confidence (Sterz et al. 2022). Making mistakes in a ‘safe environment’ will have a positive effect on clinical competence. Manikins allow students to perform clinical procedures, assess, and treat patients without fear of endangering the patient’s life or worsening the patient’s condition (Sterz et al. 2022). Although differences were observed in the present study, previous research comparing patient manikins and SPs in trauma team simulations has reported similar outcomes across clinical competence, learning success, realism and emotional response (Rutherford-Hemming et al. 2019).

Simulation-Based Education has been shown to have a positive effect on medical knowledge, comfort in performing procedures and improved performance or competence (Sterz et al. 2022). Although students were more satisfied with the SP modality, they mentioned that it added to their level of stress during a simulation. The increased stress reported by students during SP simulations warrants critical consideration. Stress and nervousness during SBE interactions are mentioned frequently in the literature (Slabber & Henrico 2022).

While heightened stress may reflect increased realism and engagement (Lateef 2010), particularly for novice learners, excessive stress has the potential to negatively impact learning if psychological safety is not adequately supported (Rudolph, Raemer & Simon 2014). First-year students, who are still developing confidence and professional identity, may be especially vulnerable to performance-related anxiety when interacting with simulated patients. However, a degree of stress may also be educationally valuable, as it mirrors the emotional and cognitive demands of real clinical encounters. These findings highlight the importance of carefully balancing realism and psychological safety in simulation design, particularly when using SPs with early-stage learners (Rudolph et al. 2014).

The usefulness of HFMs within medical education is invaluable both in terms of learning outcomes and student acceptance (Rutherford-Hemming et al. 2019). However, this study showed that the manikin did not score favourably in terms of case application, self-confidence and equipment use. Although high-fidelity simulations are designed to resemble real clinical scenarios, there is limited evidence defining the degree of manikin realism required to maximise learning outcomes (Riaz et al. 2020). Manikins are useful when practising invasive procedures like endotracheal intubation (Glosser et al. 2022).

Understanding the advantages and disadvantages of different SBE modalities will be beneficial to student learning and will allow the facilitator to appropriately implement SBE in their context. This might mitigate the lower scores received for case application and equipment-used domains, which were evident in this study.

Literature suggests that students will always slightly favour SPs as they are more realistic and allow direct communication. The SP modality enables students to practise clinical skills, including history gathering, physical examination, and the application of medical knowledge within a simulated setting in which information is obtained directly from the patient (Glosser et al. 2022). The interactions mimic the real-life physician-patient interactions in a more realistic approach (Shankar et al. 2016). This could, in turn, improve the self-confidence of students, as this is not always possible with a manikin.

The SSHF is structured around eight domains (only seven were used in this study). The SP modality scored favourably in all domains, and the manikin scored below four in two domains (domains 2 and 7). Domain 2 focused on the characteristics of SBE cases and their application. Domain 7 was concerned with the facilities and equipment used. The characteristics of SBE cases will always be different, and so will their application.

Importantly, this study’s findings offer guidance for simulation-based curriculum design in contexts where training resources must be carefully optimised. The students’ appreciation for SPs, despite less frequent exposure to this modality, suggests an unmet pedagogical need.

### Limitations

This study focused on student satisfaction regarding the use of SP and HFM modalities in pre-hospital emergency SBE scenarios at a single South African institution. As a pilot study with a small sample size, the findings are not intended to be generalisable. However, they provide preliminary insights that can inform the design and methodology of future, larger studies. The use of descriptive statistics without confidence intervals limits the precision of estimates and should be addressed in future studies with larger samples. This study also did not include formal debriefing in the scenarios completed by students. The authors acknowledge that debriefing is known to enhance reflection, learning consolidation and psychological safety in SBE. Its exclusion may have influenced students’ learning experiences and perceptions. Additionally, all participants completed the same scenario using both simulation modalities without randomisation of order. As a result, improvements in confidence may partly reflect familiarity with the scenario rather than differences between SPs and HFMs.

### Recommendations

Findings suggest that incorporating SPs more systematically into EMC curricula may enhance realism, student confidence, and readiness for clinical practice. At the same time, HFMs remain indispensable for technical skill training. A blended approach, combining both modalities, may offer the most comprehensive educational benefits. Consideration of student stress and psychological safety is essential when introducing realistic simulation modalities, particularly in early training. These insights may assist educators in optimising simulation use within resource-limited curricula. For policymakers and curriculum designers, the study highlights that investment in SPs, though resource-intensive, is justified by student satisfaction and the development of critical interpersonal competencies. In resource-limited contexts, prioritising SP integration may yield greater educational returns than exclusive reliance on HFMs.

Further research is needed to determine the satisfaction of students concerning all SBE modalities at various institutions and health sciences departments. It would also be advisable to include all second-, third- and fourth-year students.

## Conclusion

Both SPs and HFMs are indispensable in EMC education, offering unique benefits that address different aspects of clinical training. While HFMs excel in procedural practice, SPs provide the interpersonal realism necessary for holistic pre-hospital care. This pilot study demonstrates that even in small EMC student cohorts, SPs can enrich clinical simulation by offering realism, emotional engagement and communication challenges. Even in a small cohort, EMC students’ preferences highlight the importance of integrating SPs into simulation curricula. While HFMs remain indispensable for technical training, SPs offer a uniquely human element that enhances learning. These findings support a blended approach to simulation in EMC education.
